# Snake Fungal Disease in Free-Ranging Northern Pine Snakes (*Pituophis melanoleucus melanoleucus*) in New Jersey: Lesions, Severity of Sores and Investigator’s Perceptions

**DOI:** 10.3390/jof10020125

**Published:** 2024-02-03

**Authors:** Joanna Burger, Christian Jeitner, Robert T. Zappalorti, John F. Bunnell, Kelly Ng, Emile DeVito, David Schneider, Michael Gochfeld

**Affiliations:** 1 Cell Biology and Neuroscience, Rutgers University, 604 Allison Road, Piscataway, NJ 08854, USA; christian.jeitner@pinelands.nj.gov; 2 Ecology, Evolution and Natural Resources, Rutgers University, 14 College Farm Road, New Brunswick, NJ 08901, USA; kelng@dls.rutgers.edu; 3 Center for Environmental Exposures and Disease, and Environmental and Occupational Health Sciences Institute, Rutgers University, 604 Allison Road, Piscataway, NJ 08854, USA; mg930@eohsi.rutgers.edu; 4 New Jersey Pinelands Commission, New Lisbon, NJ 08064, USA; john.bunnell@pinelands.nj.gov; 5 Herpetological Associates, Inc., Pemberton, NJ 08068, USA; rzappalort@aol.com (R.T.Z.); dschneider@herpetologicalassociates.com (D.S.); 6 New Jersey Conservation Foundation, Far Hills, NJ 07931, USA; emile@njconservation.org; 7 Rutgers Biomedical and Health Sciences, Rutgers University, Piscataway, NJ 08854, USA

**Keywords:** snake fungal disease, *Ophidiomyces ophidiicola*, visual evaluation of sores, pine snake

## Abstract

*Ophidiomyces ophidiicola*, the fungus causing snake fungal disease (SFD), has been identified in northern pine snakes (*Pituophis melanoleucus*) in New Jersey. In this paper, we (1) review the positivity rate of SFD on different locations on snakes’ bodies, (2) determine the relationship between the sores and quantitative polymerase chain reaction (qPCR) positivity rates, and (3) explore the relationship between the investigators’ clinical evaluation of the severity of sores, their evaluation of the likelihood of the sores being positive, and the qPCR positivity of SFD for the sores. Swabbing the sores was more effective at determining whether the snakes tested positive for *O. ophidiicola* than ventrum swabbing alone. The perception of the severity of the sores did not relate to qPCR positivity for *O. ophidiicola*. We suggest that the assessment of the rate of SFD among snakes in the wild needs to include the sampling of snakes with no clinical signs, as well as those with sores, and the swabbing of all the sores collectively. Clear terminology for sores, the identification of clinical signs of SFD, and distinguishing the rates of *O. ophidiicola* by PCR testing should be adopted. Overall, the pine snakes exhibited a higher rate of sores and positivity of *O. ophidiicola* swabs by PCR testing compared to the other snakes.

## 1. Introduction

Fungal diseases have the potential to cause sublethal effects, lethal effects, and to devastate populations of vertebrates [1,2,3]. Even though habitat fragmentation and loss, illegal exploitation (“poaching”) and climate change may devastate snake populations, fungal diseases may also play a significant role in local snake declines and possibly local extinctions [4,5,6]. Ophidiomycosis, also called snake fungal disease (SFD), is caused by *Ophidiomyces ophidiicola* [7]. SFD can cause severe disease and mortality in some snake species [4,8,9,10]. Laboratory experiments have demonstrated that SFD is caused by *O. ophidiicola* by showing that snakes experimentally infected with the fungus develop skin lesions (=sores) and other abnormalities observed in the wild and that the fungus was isolated from infected snakes in the wild [11,12,13]. McKenzie et al. [14], in laboratory experiments of *O. ophidiicola*-inoculated snakes, found that only 21% of the skin swabs (not sores) tested positive for SFD DNA despite all the live specimens testing positive. Although the incidents of SFD and the recognition of SFD began increasing around 2013 [9], Lorch et al. [15] and others [16,17] demonstrated that *O. ophidiicola* has been present in wild snake populations in the U.S. for decades.

Although it was recognized in captive snakes before 2000, the first confirmed North American case of SFD in a wild snake was in a Eastern Massasauga rattlesnake (*Sistrurus catenatus*) in Illinois in 2008 [17], and later in Michigan [8]. SFD has since been confirmed in many other wild North American snakes [18,19,20,21,22]. Further, Lorch et al. [15] recently reported confirmed cases of Ophidiomyces in museum specimens collected as early as 1945, including in timber rattlesnakes (*Crotalus horridus*), corn snakes (*Pantherophis guttatus*) and milk snakes (*Lampropeltis triangulum*). SFD has been reported in several snake families in North America, most commonly in Colubridae and Viperidae and in semiaquatic species [9] as well as terrestrial snakes [23,24].

Reports are continuing to appear concerning the symptoms, diagnostics, prevalence, mortality and effects on populations, as well as new hosts and new locations [4,22,25]. The apparent range extensions may be real or indicative of increased awareness and efforts, or both. Developing recovery strategies that enhance detection and minimize the disease prevalence at the population level may be essential to achieve population stability for some vulnerable species, especially given that prevalence and mortality vary greatly among species [8,21,26]. Further, the SFD rates are higher in the winter than in the summer [27,28] and are likely more common in hibernacula [29] and just after leaving hibernation [30].

Sampling snakes for the presence of the fungus is also methodologically challenging. Several authors have noted that *Ophidiomyces* is associated with sores (=lesions) and have noted that swabbing is the best non-invasive method of determining whether the fungus is present in a sore. McKenzie et al. [24] reported that clinical signs (e.g., epidermal flaking and crusting, dermatitis, discolored scales, skin lesions, facial swelling or discharge) were a strong predictor of *O. ophidiicola* presence, as determined by a positive quantitative polymerase chain reaction (qPCR) test. Skin swabs, followed by qPCR, are an effective method for the detection of *O. ophidiicola* DNA, in association with clinical signs [21,31]. Using swabs clearly reduces stress and the handling times compared with those of biopsies. However, biopsies offer the opportunity for histopathologic as well as genetic identification. qPCR detection is used to identify SFD [21]. 

Some authors have found *O. ophidiicola* in or around the sores, abnormal scales, superficial swellings and sub-cutaneous swellings on the dorsal surface of snakes, as well as the ventrum and sides [32]. However, the methods of swabbing and the attention given to the sores (type and location) can affect the diagnosis, detection, and the classification of whether a snake is positive or not for SFD [21]. Several methods of evaluating the clinical signs have been developed [33,34]. In several studies, snakes without any sores yielded positive *O. ophidiicola* DNA, suggesting there is an asymptomatic carrier state, and there are clearly hotspots of SFD infections [6,34]. The detection of the fungus in a sore is not proof that the fungus caused that sore because sores may occur naturally in some snakes, with the fungus opportunistically colonizing the damaged tissue [9]. 

We have been studying the behavior and ecology of pine snakes, *Pituophis melanoleucus melanoleucus*, around hibernacula since the 1980s, which includes PIT tagging, measuring and examining each snake at the end of hibernation [35,36,37]. In our experience, pine snakes often have “sores” in the form of raised, damaged or discolored scales, notched or ragged scales, and less often, mounds of discolored, deformed or degraded scales, which are easily dislodged. We called these “hibernation sores,” and assumed they were a minor affliction probably associated with soil dampness, trauma or abrasions while digging their hibernation chambers. We saw no evidence of morbidity or mortality related to disease or starvation among the hibernating snakes [36,37]. “Hibernation sores” are recorded in our notes from our earliest work with the pine snakes. Pine snakes shed several times a year, which heals most sores. We found most of these same snakes in hibernacula in subsequent years [37], and even the chronic sores were sometime gone the following year.

In this paper on northern pine snakes from the New Jersey Pine Barrens, we examine (1) the prevalence or positivity rate of SFD (determined by qPCR) on the head, ventrum and dorsal surfaces, cloaca, and obvious sores (12 snakes from 2018; 72 snakes from 2019 to 2021), (2) prevalence as a function of age, (3) the relationship between the sores and qPCR positivity rates (2019 and 2020), and (4) the ability of the researchers to clinically evaluate the severity of the sores and identify the likelihood that a particular sore would test positive for SFD (2019–2020). In this study, qPCR served as the Gold Standard for diagnostic accuracy. 

## 2. Materials and Methods

### 2.1. Study Species and Study Sites

Our long-term studies of the ecology and behavior of the northern pine snake began in the late 1970s. We began excavating hibernacula in the mid-1980s. In New Jersey, pine snakes emerge from hibernation in late March–April, mate in April–May and nest in late June–early July. Hatchlings typically emerge in early September, and they must find a hibernation site [37]. In the fall, many hatchlings reach the communal hibernacula by following scent trails [37,38,39]. A snake that hatched in September is designated as age 0 in our database when encountered in the fall of its hatching year. The same snake, encountered the hibernacula when they were excavated in early March, is designated as one-year old. The following March, this snake (ca. 18 months old) is designated as a two-year old. We separate the 1-year-olds because they enter the hibernacula free of SFD in the fall (*O. ophidiicola* negativity was determined by swabbing 41 hatchlings in the fall) [29]. 

We currently work at three “hibernacula complexes” of 3 to 5 dens each in Burlington and Ocean County, New Jersey. We do not divulge the exact locations of these dens due to high levels of poaching [36]. Each active den has from 1 to 15 pine snakes, depending upon the year. Our field and laboratory studies are approved by Rutgers University Animal Care and Use Committee (permit # E6-017, renewed every three years) and by permits from the New Jersey Department of Environmental Protection (Endangered and Nongame Species Program, renewed every year) and New Jersey Parks and Forestry, and with permission from private landowners. In the design and execution of our studies, the snakes’ welfare is always our greatest concern.

We initiated a pilot study of SFD of 12 individuals in 2018 using careful field hygiene procedures to avoid introducing or spreading the fungus if it was present. Based on the surprising positive results (58% positive detections), we launched a more detailed study in 2019. Our overall approach thereafter was to examine the prevalence of *O. ophidiicola* detection in all the free-ranging snakes located in our excavated hibernacula in 2019, 2020 and 2021. We collected swab samples from the snakes as they were removed from the hibernacula in early spring before emergence. 

### 2.2. Sample Collection

Our overall protocol was to remove the snakes from their hibernation site, examine them for sores and swab them for SFD immediately, and then process the snakes. Processing included measuring and weighing the snakes and inserting a PIT tag if they were new. All personnel handling the snakes changed nitrile gloves between each snake. In the 2018 pilot study of 12 pine snakes, only the entire ventral surface from the head past the cloaca to the tail tip was swabbed in a single pass. In 2019–2021, we collected ventrum swabs as well as swabs of the head and cloaca and a separate swab for each sore for all the snakes.

**Figure 1:**
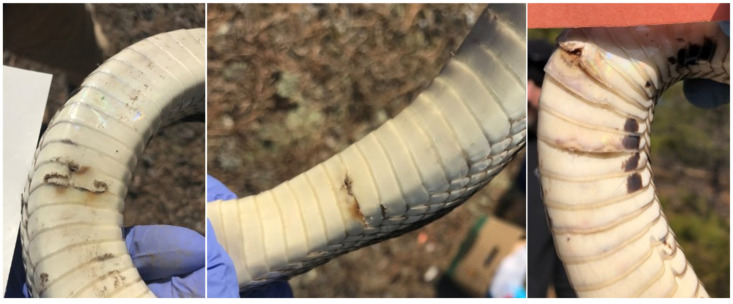
Examples of sores. Typical sores on pine snakes involve discolored or jagged scales, abraded scales, crusting and other gross clinical signs.

For each snake, we swabbed each different part of the body with sterile polyester tipped swabs that were premoistened with sterile deionized water. We sampled the ventral surface using a swab from the head to the tip of the tail, excluding any lesions present [29]. Additional swabs were taken of the head and from each individual sore (=lesion, Figure 1). For the purposes of swabbing, a sore included any raised, damaged or discolored scales, notched or ragged scales, and less often, mounds of discolored, deformed or degraded scales. The swabs were stored in screwcap tubes, placed on ice in the field and stored frozen at −30 °C in a freezer until analysis. 

We use the term “sore” very broadly. There were rarely obvious abrasions, crusting, scabs or raised scales on the heads of the pine snakes, or mounded scales or ulcers on the dorsal surface. Most sores were on the ventral surface of the snakes on, under the scales, or as scales with discolored ragged or jagged margins, conspicuous against the immaculate white ventral surface (Figure 1). Some discolorations extended across or under several ventral scales. Because of the controversy about whether the sores are caused by *O. ophidiicola*, or whether the fungus is an opportunist invading sores with or without active infection [9], we examined the sores in more detail. In some years, the pine snakes had many sores. In others, there were few. Individual snakes can have one or more sores. For this study, we define a sore as any recognizable skin or scale abnormality, whether severe (abrasions, lesions, breakages, or discoloration covering multiple scales or even the whole ventrum) or minor (slight discoloration or a raised scale). All the sores in 2019–2020 were tested using PCR for *O. ophidiicola*, numbered and photographed.

### 2.3. Identification of SFD by qPCR

The presence of *O. ophidiicola* was determined by the extraction of nucleic acid from swab samples using a specific qPCR targeting the internal transcribes spacer region of the fungus as described by Bohuski et al. [40]. The samples were defined as positive for *O. ophidiicola* if they had 15 or more copies of target DNA (based on standard curves on each PCR run). A snake was considered positive in this paper if it was PCR positive for any one of the swab samples (e.g., ventrum, head, sore or cloaca). In this paper, we assume that, with PCR, there are very few false positives, which may arise from sample contamination. False negatives can arise from the sampling technique in the field or sample handling before or after reaching the laboratory. Some of the variabilities noted may be due to such test errors. Further information on methodology can be found in Burger et al.’s study [29] and Bohuski et al. [40].

### 2.4. Investigator Evaluation of Sore Severity and SFD

At the end of three seasons of sampling for SFD and identification through qPCR [29], we tested the use of clinical signs (discoloration, scabbing and size) to determine the severity of a sore and the investigators’ perceptions of whether the sore was likely to be positive by qPCR or not. In other words, could the investigators working with snakes that tested positive for SFD predict whether a given sore was positive by PCR or not based on their evaluation of the severity of the sore (e.g., its appearance)? The 2018 sample did not include sores, so the following is based on the snakes swabbed in 2019 and 2020. The photographic cards with snake sores that were scored for severity were later evaluated for clinical diagnosis after the photographic cards had been shuffled. The steps in the process involved (1) taking photographs of every sore noted on every snake from our hibernacula in 2020 and 2021; (2) printing the photographs of sores (with their field identification numbers); (3) having each author (except K. Ng) score each sore on each photograph for severity (Severity Score) on a scale of 0–5, where 5 was most severe; (4) reshuffling the photographs, and then having each person determine whether each sore was likely to be PCR-positive or PCR-negative; and later, (5) analyzing the results. Each participant (n = 7) completed both the severity and positivity test independently in separate sessions in a quiet room without coaching. All the evaluations of sores were conducted after the end of hibernation in 2021. All the investigators that evaluated the sores had worked with pine snakes (as well as other snake species) for over 10 years and were involved with SFD studies for 4 years, paying particular attention to clinical signs and sores. The results were later compared to the qPCR results for each sore. Since 7 researchers evaluated the severity of each sore, we computed the mean severity for each sore. For the diagnostic test, if 4 or more researchers determined a sore as “likely positive”, it was considered positive. All evaluations were performed independently several months after the last snake had been photographed, and only the portion with sores was visible (e.g., individual snakes could not be identified).

### 2.5. Statistical Analysis

We used non-parametric procedures (Kruskal–Wallis X^2^ test or Fisher’s exact test, PROC NPARIWAY) to determine the differences among the swabbing locations and among the sores [41,42,43]. These non-parametric tests were used because they are more conservative and are best suited for small datasets with binary outcomes [41,43]. The Severity Assessment resulted in a score for each sore as mean of the 7 observers. The Severity Assessment was based on 93 sores (not all snakes sores had usable photographs). The mean scores for PCR-positive and PCR-negative sores were compared with a Kruskal–Wallis test. Diagnostic Assessment was performed to determine whether a sore would likely be PCR-positive or -negative, and the data were tested for sensitivity (correct identification of positive lesions) and specificity (correct identification of negative lesions) according to the framework for evaluating diagnostic test accuracy [44]. *p* values of 0.06 and below were considered significant, given the relatively small sample size [41,42,43]. 

## 3. Results

### 3.1. Positivity Rate of SFD as a Function of Age, Gender and Different Locations on the Snake

If the snakes had any positive qPCR test, they were considered positive. Thus, some snakes did not have a positive ventral swab, but did have a positive test on the head, sore or cloaca. Overall, 73% of the pine snakes (n = 84) had at least one positive qPCR test for *O. ophidiicola* DNA.

**Figure 2:**
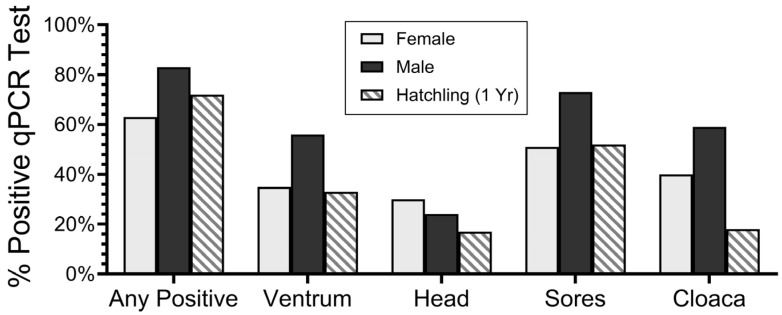
Percent of pine snakes that tested positive overall (any positive test) by location and by males versus females compared to hatchlings (after Burger et al. [29]). Graph is based on 84 snakes for any positive qPCR test and 72 snakes for head, ventrum, skin/scale lesions (sores) and cloaca.

The positivity rate of SFD varied by age, gender and location on the snake (Figure 2). A significantly higher percentage of males over 1 year of age tested positive for SFD than the females did for the ventrum, sores and cloacal swabs (Fisher’s exact tests were all *p* < 0.05). However, the percentage of hatchlings that tested positive for SFD did not differ significantly from that of the adults overall, and there were no sex differences among the hatchlings (Fisher’s exact test). These data provide the background for the examination of the data in this paper; additional information is provided in Burger et al.’s study [29]. 

### 3.2. Relationship between Sores and qPCR Positivity Rates

**Figure 3:**
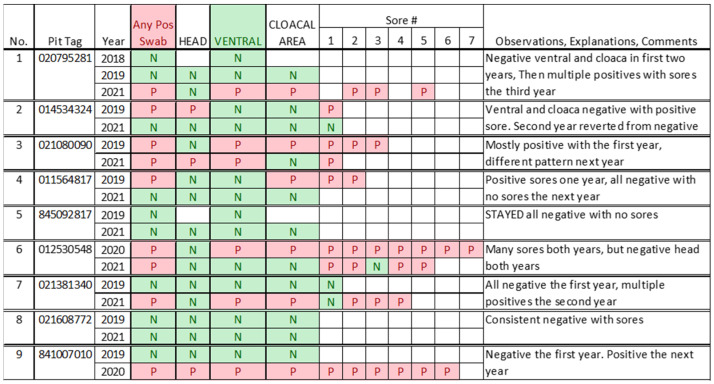
Examples of distribution of *Ophidiomyces ophidiicola* detection by PCR between years in individual snakes. These were selected to illustrate several points, including change in positivity between years, and variability in the positivity of sores on individual snakes. If a box under sore is empty, it means there was no sore. The sores in subsequent years are not the same sore, but are new ones. P = Positive PCR test for SFD and coded red, N = Negative PCR test for SFD and coded green.

The variability in positivity rates as a function of different sampling sites on the body (and different sores) is illustrated in Figure 3. This figure shows whether the individual had any positive tests, the positivity and negativity of different sampling sites, and the variability among the different sores on the snakes that had sores. These examples were selected to illustrate different patterns, and the complexity of prevalence, including variation from one year to the next. Individual variations by year will be addressed in the future. The snakes can test from negative to positive the following year, or vice/versa. Some snakes had up to seven sores, and some that were entirely negative one year had been positive the previous year (e.g., snake 4). This also illustrates that the situation with nearly every snake is unique. Some snakes had almost all positive sores, leading us to question whether the isolated negative sores are true negatives. The other snakes had all negative sores, which we believe are true negatives. These data indicate the need to follow the individuals for several years to understand the long-term effects of SFD in pine snakes. The prevalence of PCR positive sores was 72% in 2019, 83% in 2020, and 70% in 2021 [29].

Sores can test positive or negative for a fungus using PCR. A snake can have only one positive sore and multiple negative sores, or vice versa. Table 1 summarizes the relationship between the sores and *O. ophidiicola* detection. Table 1 shows (1) a marginally higher proportion of two-year-old and older snakes that had at least one sore, (2) no age-difference in the proportion of snakes with at least one positive sore, (3) no difference in the percent of swabbed sores that were positive, and (4) no difference in the mean number of sores or positive sores per snake with any sores.

In the following sections, we examine the relationship between the researchers’ evaluation of severity of a sore and PCR positivity (Section 3.3); whether the researchers thought a sore was positive or not (Section 3.4); and evaluations combining the Severity Scores, yes/no perceptions of the researchers and the PCR test results (Section 3.5).

### 3.3. Relationship between Evaluation of Sore Severity Scores and Positive qPCR Tests

**Figure 4:**
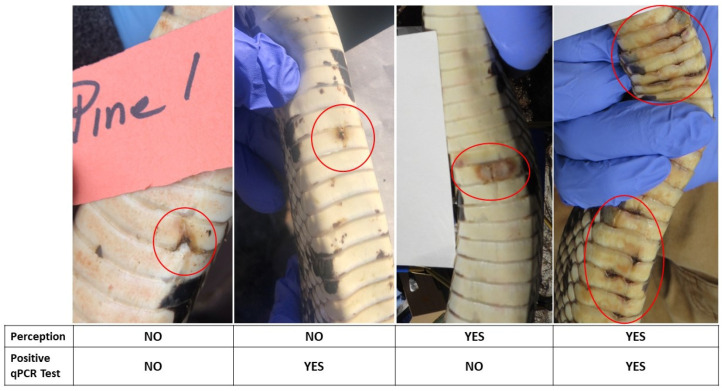
Examples of sores that were evaluated by seven researchers for whether they would likely test positive or negative for SFD by qPCR. If the majority of researchers called the sore “likely positive”, it was counted as positive.

One of the main objectives of this paper was to compare the investigators’ evaluations of the severity of the sores (on a scale of 0–5) and their ability to identify whether a sore was likely to be PCR-positive or -negative for *O. ophidiicola* detection (Figure 4). Only the swabs collected in 2019 and 2020 were used because the sampling procedures (and personnel) were similar. The participants were aware that more than half of the pine snakes we tested in 2018 were positive for *O. ophidiicola,* but did not know the positivity rate for the other years. Although mean Severity Scores did not differ between the snakes that tested negative or tested positive for 2019, the mean scores did differ for 2020 (Table 2). However, surprisingly, the mean Severity Score was significantly higher for the sores that tested negative for *O. ophidiicola* detection using PCR.

### 3.4. Relationship between Diagnosis and a Positive qPCR Test

The photographic cards of the snake sores that were scored for severity were then evaluated for a clinical field diagnosis (yes/no for SFD) after the cards with the photographs had been shuffled. Each of the seven researchers determined whether they thought the sore would likely test positive or negative by PCR (=diagnosis). If four or more labelled it as “positive”, it counted as a positive diagnosis. When the researchers determined that a sore was likely positive, they were right (true positive) 58% of the time. Because of the high prevalence (80%), the negative predictive value was only 16%, while the positive predictive value was 77%.

Figure 4 presents some examples of the perceptions (evaluations) by the researchers of the sores. The examples were selected to illustrate the relationship between the appearance of sores and a positive PCR DNA test for *O. ophidiicola*. The photos illustrate that the Severity Sores did not always agree between the researchers’ evaluations and a positive PCR test.

The challenge to correctly diagnosing the SFD-positive PCR sores lends itself to the framework of evaluating the accuracy of diagnostic testing, which is the basic principle of epidemiology, as laid out by Korevaar et al. [44] in Table 3. Each sore had a diagnosis as positive (if four or more investigators labelled it positive) or negative for SFD. The PCR test serves as the Gold Standard for SFD.

Table 3 summarizes the observers’ performance at reaching a diagnosis of SFD. At this stage in our experience with SFD (2021), our ability to correctly diagnose an SFD-positive sore was barely better than doing so by chance (e.g., 50%; Sensitivity was 58%). We did even less well identifying a sore as “negative”, perhaps because of the low overall prevalence of negativity (20%). Once we did identify a sore as probably positive, we were right ¾ of the time (positive predictive value = 77%). The negative predictive value (16%) suffered from the low overall prevalence of PCR negative sores. These determinations assume that the Gold Standard is always accurate. The positive predictive value is very sensitive to the prevalence, performing much better when a disease is common than when it is rare. 

### 3.5. Severity Scores, Diagnosis and a Positive qPCR Test

**Figure 5:**
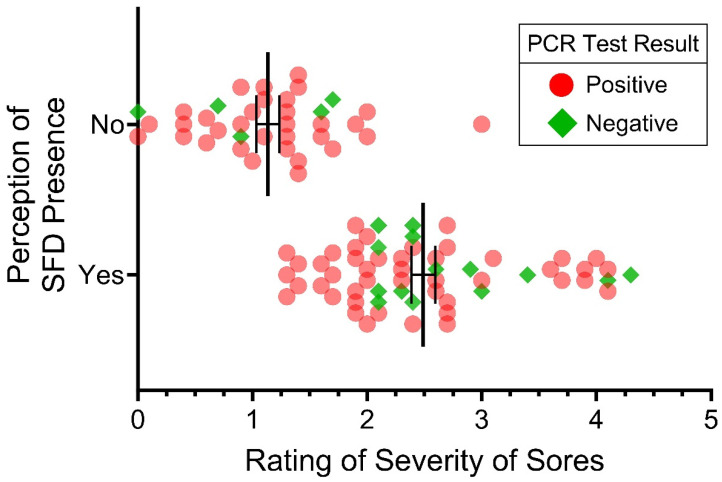
Identification (=diagnosis) of SFD sores (yes vs. no) in relation to SFD sore Severity Scores for pine snakes swabbed in 2019 and 2020 near the end of hibernation. Red dots indicate a positive PCR test (n = 74). Green diamonds represent a negative PCR test (n = 19). Sores with higher Severity Scores were more likely to be called “positive”, particularly in 2020. Vertical lines represent the mean scores and standard error.

We compared the perceptions of severity averaged over seven participants, with the actual qPCR test results for each sore (Figure 5) for the 2019–2020 samples. For this figure, we computed the mean severity score for each sore and plotted the mean scores, comparing the positive and negative qPCR results. Overall, the sores that tested negative for SFD had lower mean scores than those that tested positive. However, this figure also demonstrates that the sores that tested negative are scattered throughout the graph. That is, the perceptual severity of a negative score could be rated anywhere from zero to five (a few sores were rated as five by all the researchers). The scores for the positive sores were less variable.

### 3.6. Sampling Techniques: Clinical Signs and PCR Testing

The study of any infectious disease requires consideration of the sampling methods that adequately identify the presence of the disease organism and the reliability of that method. In the case of SFD, prevalence can be determined by clinical visual examination (either in the field or laboratory), or by measuring *O. ophidiicola* by PCR or culturing *O. ophidiicola* [21]. Both later methods require swabbing a snake or biopsying the sores. The swabbing and biopsy results for *O. ophidiicola* are highly correlated [21]. There are at least two questions related to these techniques: (1) Where and how does one swab a snake or perform a biopsy? (2) How many swabs or biopsies should one take? Another key issue is whether to swab every snake encountered, or only those that show clinical signs of sores, or other dermatitis conditions.

We suggest that if the snakes have sores, clearly, they should be swabbed. That is, 82% of the sores of the 1-year-olds tested positive by qPCR, and 80% of the sores of the snakes older than 1 year tested positive (Table 1). However, if there are no sores on a snake, and funds are limited, the question arises as to what protocol to follow in terms of the samples to collect. Taking only those snakes in which at least one swab tested positive, Table 4 shows the percentage that each swab location tested positive for the 1-year-old and older snakes. The best prediction of positivity came from swabbing the sores (about 80% were positive). However, if there were no sores, the highest rate of predictability of a positive qPCR diagnostic for the other sampling swab techniques was for the ventrum and cloaca compared to the head in pine snakes. The older snakes had higher positivity rates than the 1-year-old pine snakes (Table 4).

## 4. Discussion

### 4.1. Sampling Methods

In our pine snake studies that began in the 1970s, we often encountered sores involving discolored or deformed scales, with the flaking or swelling of the skin. Until 2018, we dismissed these as “hibernation sores” and not serious because some of the same snakes were found in the hibernacula in the following year, often without these sores. After 58% of the ventrum swabs of 12 pine snakes sampled in 2018 tested positive for *O. ophidiicola*, we initiated a more in-depth study. In 2019 and 2020, we took swabs of the head, ventrum and cloaca for all 72 pine snakes encountered (some snakes were sampled in both years). We also swabbed each sore. In the subsequent years, we found that the ventrum swabs were often negative in the snakes that were positive for the other swabs (Figure 2), particularly of the sores. Positive ventrum swabs were the sole positives in only two snakes (Table 4). Our findings corroborate the findings of many others that ventrum swabs alone are not adequate for assessing the prevalence of *O. ophidiicola.* That is, collecting only one swab from a snake can result in false negatives. Davy et al. [25] also suggested that swabbing in general has a high false negative rate. Hilemann et al. [31] reported that swabbing with only one applicator swab resulted in a high probability of false negatives in the snakes with clinical signs of SFD. 

True negative swabs may occur in snakes with localized infections. Moreover, differences in detection can result from seasonal or species differences in the host clinical presentation or field/lab methodologies [28,31,45,46,47]. Some authors swab only the sores, some swab ventrally once, while others swab ventrally and intentionally swab the sores vigorously [47]. Thus, comparisons among the studies are difficult. Allender et al. [8] noted that snakes with clinical signs can test negative for SFD. For example, only 85% of pygmy rattlesnakes with clinical signs were positive for *O. ophidiicola* DNA [45]. Some researchers have found that clinical signs of SFD can serve as reliable indicators of *O. ophidiicola*. in the field [24,28]. Others categorize snakes as infected if they tested positive for *O. ophidiicola*, or, in the absence of pathogen detection, had clinical signs consistent with SFD previously reported for that species [11,17]. Chandler et al. [48] noted that all Eastern indigo snakes that tested positive had skin sores, but they classified the additional snakes as having sores consistent with SFD that they did not test. In our study, 20% of the sores of the pine snakes did not test positive for *O. ophidiicola*. These data suggest that several sampling methods may be essential (along with PCR testing) to accurately reflect SFD caused by *O. ophidiicola*. Parenthetically, Vivirito et al. [49] recently suggested that ultraviolet fluorescence can be used as a field-applicable screening tool for sores that is consistent with Ophidiomycosis in watersnakes (*Nerodia sipedon insularum*). Fluorescence was highly associated with the skin sores, and one skin sore was 100% specific for identifying watersnakes that tested positive for Ophidiomycosis [49]. We note in passing that we also found *O. ophidiicola* in some of the soil samples below the hibernating pine snakes [50]. Three other questions still remain: (1) If an external swab is negative, is it a true negative? (2) If an external swab is positive, does the snake have *O. ophidiicola* internally? (3) If a swab is positive, does it impair (or kill) the snake? Lastly, of course, can field researchers correctly identify a sore as positive or negative? The answer is no.

Determining whether a negative swab is a false negative or reflects the true absence of the fungus in the swabbed area can be partially determined by replicate swabbing. These challenges argue for clear definitions of terms (including what constitutes a positive case of SFD for snakes), the sampling of all the clinically defined abnormalities, and the standardization of the sampling techniques to clarify the presence of *O. ophidiicola*. We suggest, that the researchers distinguish between the snakes that test positive for *O. ophidiicola* or not, have sores or not, whether the sores test positive or not, and whether the snakes without any clinical signs test positive. The numbers of snakes (and multiple sampling of individuals) requires careful documentation. Age, sex, season and year may contribute to the variability. Determining whether a positive sore is caused by the fungus (i.e., SFD), or whether the fungus represents a secondary infection is important as well. Our ventrum sampling involved running a moistened swab firmly down the ventral surface from chin to just anterior to the cloaca. However, we sampled the sores more aggressively, inserting the swab under or between the scales. 

### 4.2. Limitations of the Study

The objective of the study was to determine the best location to test the pine snakes for SFD and to see whether field personnel familiar with both pine snakes and SFD could predict whether a sore on a pine snake was indicative of a positive PCR test. Seven investigators familiar with pine snakes evaluated the severity of a sore and whether they felt it would test positive for SFD with a PCR test. The possible limitations of this study were (1) more investigators could have been sampled, (2) guidelines or descriptions of the scoring could have been provided, and (3) the sample sizes of the sores could have been larger. The investigators selected had all worked with pine snakes for over a decade and with SFD for 4 years, and thus, they had more experience with pine snakes than most field personnel might have. This meant that more naïve personnel might have had more difficulty with the task. While the sample sizes were small, they are relatively large for a sample of snakes collected at the same time of the year in similar conditions (e.g., hibernation). Finally, descriptions were not provided because the objective was to obtain information and evaluations from field personnel without biasing their responses. Developing a rating scale of the severity of sores is a different project and is worth developing. Yet, the data in this paper indicate that the severity of the appearance of a sore was not indicative (or predictive) of whether the sore would test positive using q-PCR. 

### 4.3. Perception and PCR Testing

The utility of the field diagnosis of sores as SFD or not SFD is an important consideration, particularly in determining the geographic or taxonomic spread of the fungus or in assessing individuals. Experience, practice and test confirmation should improve the individual and collective ability to identify the disease. The literature describes a variety of sores. Our first attempt to score the severity of sores based on the 2019–2020 samples showed some difference among the years. The ability to make a correct diagnosis was less impressive. Based on 93 test cards, our ability to correctly diagnose the SFD positive sores was barely better than doing so by chance. Once we did identify a sore as “probably positive”, we were right ¾ of the time. Once the prevalence in a population becomes known, the observers will incorporate that information into their diagnoses.

The relationship between the evaluation of clinical signs and PCR testing is partly a matter of perception. Several papers have published and evaluated the clinical signs of “disease” in snakes, and subsequently, the tests for *O. ophidiicola*. In some cases, the snakes tested positive, and some did not. Like our studies, Lind et al. [28] assigned a score (1–3) to Ophidiomycosis signs in pygmy rattlesnakes using only snakes with one clear skin sore, and found 78% positivity. We used a similar score (0–5) and found similar results. In the pine snakes in our study (not including hatchlings), 85% of the snakes with at least one sore tested positive for *O. ophidiicola*. This is a high level of agreement, but again, it relates only to the snakes with at least one skin sore.

However, in our sample (snakes from 3 years of samples at the end of hibernation), 45% of the 1-year-olds and 24% of those over 1 year had no sores. Of the snakes with no sores, 22% of the 1-year-olds and 35% of the older snakes tested positive. This suggests, as is shown in Figure 5, the perceptions of clinical signs in the same snake can differ among observers, and more importantly, are not always congruent with the positive PCR tests. We believe this represents a clear field identification issue, as we found that it was particularly difficult to determine whether a discoloration would test positive or not.

### 4.4. Does SFD or O. ophidiicola Cause Effects, and Is It Endemic in Pine Snakes?

Davy et al. [25] reviewed much of the literature on SFD over the last 10 years for Canada, supplemented with additional field data, and found that SFD pathology was detected in most sites despite limited or haphazard sampling. They concluded that SFD was likely an endemic rather than a novel pathogen and that it may not pose an imminent threat to most snakes. Our data on pine snakes, albeit limited to the Pinelands of New Jersey, support this conclusion; SFD is currently not presenting an immediate obvious threat to pine snake populations. At present, the populations of pine snakes in our study do not appear to be adversely affected by the presence of SFD. In the case of pine snakes, the “hibernation sores”, like those that we found positive recently, were present in the late 1970s when we first started studying pine snakes. Most of the sores on the pine snakes tested in 2019–2021 (79% of 106 sores) were positive, meaning that 21% did not test positive. In contrast, Haynes et al. [22] found that only half of the sores tested positive for *O. ophidiicola* DNA (summarized for 786 individuals of 34 species); only 27.5% had skin sores, and 13.3% tested positive for *O. ophidiicola*. 

Fungal diseases may cause other significant sublethal effects besides deformities and death. For example, Lind et al. [45] reported lower testosterone levels in infected males compared to uninfected males during spermatogenesis in the fall breeding season in pygmy rattlesnakes. McKenzie et al. [13] reported changes in behavior within a season for two species of wild snakes; there was an increased surface activity of diseased snakes. In cold climates, we suggest this could lead to the SFD snakes staying out longer than others, exposing them to freezing. Tetzlaff et al. [27] similarly reported that SFD-infected, free-ranging Massasauga rattlesnakes moved less and were less visible than the non-infected snakes, and the data indicate that the infected snakes were still basking when the others were already in hibernation. If similar effects occur in pine snakes, it may increase their exposure to predators and poachers, which indirectly affects the survival, but this requires field testing. 

The relationship between the “hibernation sores”, SFD, sublethal effects and mortality bears further examination. The mortality rate was 40% in cottonmouths inoculated with *O. ophidiicola* [11]. However, these had been inoculated, rather than having acquired perhaps a lower load of *O. ophidiicola* from another snake or the soil [50,51], in the case of pine snakes in our hibernacula Further laboratory experiments with different exposures might shed light on the susceptibility of the individuals and species to SFD, but we suggest the inoculation should be dermal, as occurs in nature. Although these hibernation sores in NJ pine snakes are like those identified as SFD in other species, it is uncertain whether the fungus initiates the scale or cutaneous damage, or is an adventitious “visitor” that collects on or under damaged tissue (for example, abrasions from mammal bites or abrasions due to their extensive digging behavior). 

Rajeev et al. [52] noted that the fungus shows restricted growth below 15 °C, but *O. ophidiicola* grows in a laboratory at 5–35 °C [11,21]. Pine snakes are clearly subjected to SFD and temperatures well below this in hibernation sites, as the soil temperatures can reach 3 °C in the hibernacula. In 38+ years of the examination of pine snakes in several hibernation sites, we have never found any mortality that was not directly related to freezing (a female and two hatchlings that went down into a hibernation site too late and froze less than 0.5 m below ground), predation (a live shrew actively eating three pine snakes), or compression (a 35 g hatchling squashed by a 1200 g pine snake laying over it (Burger, pers. obs.)). Nor have we found snakes with severe head sores or ulcerated sores, or those that have a low body weight in hibernation (1986–2022). 

## 5. Conclusions

In our pine snake study, ventrum swabs alone were not as effective at determining whether the snake tested positive for *O. ophidiicola* as swabbing sores, although the sores on the same snake sometimes differed in positivity. Head sores, prominent in many SFD reports, were seldom noted in our pine snakes, but the head swabs were only positive in 27% of the snakes, mostly with no evident sore. The perception of the severity of a sore did not relate to whether it tested positive for *O. ophidiicola*. The sores identified as likely positive had higher scores than those identified as likely negative. Only 58% of the sores identified as likely positive were PCR-positive. We suggest that the assessment of the rate of SFD among the snakes in the wild needs to include the sampling of snakes with no clinical signs as well as those with suspect sores. Sore severity was only slightly useful. For cost efficiency, all the sores could be sampled with a single swab, yielding data on whether the individual snake is positive. Further, clear terminology for the sores, the identification of clinical signs of SFD, and distinguishing the rates of *O. ophidiicola* by PCR testing should be adopted. Overall, the pine snakes exhibited higher rates of both sores and positive *O. ophidiicola* detection by PCR compared to those of the other species. The pine snakes were tested at the end of hibernation, when they are still hibernating, illustrating the high rates of SFD in snakes during hibernation.

## Figures and Tables

**Table 1:** Relationship between snakes, sores and *Ophidiomyces ophidiicola* PCR detection for one-year-olds and snakes that were two years and older (includes snakes from 2019–2021, unless otherwise noted).

Parameter	One-Year-Olds	Two-Year-Olds and Older	Comparison ^b^
Sample size (snakes tested)	20	72 ^a^	
Number of snakes with at least one sore (%)	11 (55%)	55 (76%)	Fisher Exact *p* = 0.06
Number of snakes with no sores	9 (45%)	17 (24%)	
Number of snakes that had at least one positive sore	10 (50%)	44 (61%)	Fisher Exact *p* = 0.02 Significant
Number of snakes with sores that were not positive	1 (5%)	11 (15%)	
Mean number of sores/snake that had sores	2.00	2.42	t = 1.10. *p* > 0.2. Not significant
Mean number of positive sores/snake with sores	1.80	2.41	t = 1.25 *p* > 0.2 Not significant
Total number of sores per age class	22	133	
Total sores that were positive	18 (82%)	106 (80%)	Fisher Exact *p* = 0.80 not significant

^a^ = Does not include the 12 snakes in the 2018 pilot, where sores were not individually swabbed. ^b^ = Presence/absence tested with a Fisher Exact 2 × 2 Contingency Table, while comparison of numbers used Kruskal–Wallis (KW) non-parametric analysis of variance.

**Table 2:** Relationship between the mean visual rating (Severity Score) as a function of the qPCR test for *O. ophidiicola*. The years are presented separately; when combining all the sores for both years, there was a slight difference (X^2^ = 4.0, *p* < 0.05). NS = not significant.

	PCR-Pos	PCR-Neg	Kruskal–Wallis X^2^
2019			
Number of sores scored	25	7	
Mean Visual Score	2.18 ± 0.15	2.14 ± 0.4	0.04 (NS)
2020			
Number of sores scored	49	12	
Mean Visual Score	1.69 ± 0.14	2.36 ± 0.32	4.6 (0.03)

**Table 3:** Framework for evaluating accuracy of diagnostic test [44] using data from 2019 and 2020.

	PCR Test Positive (n = 74)	PCR Test Negative (n = 19)	Predictive Value
Diagnosed as Positive	True Positive TP = 43	False Positive FP = 13	Positive predictive value TP/(TP + FP) 43/56 = 77%
Diagnosed as Negative	False Negative FN = 31	True Negative TN = 6	Negative predictive value TN/(TN + FN) 6/37 = 16%
	Sensitivity TP/(TP + FN) 43/74 = 58%	Specificity TN/(TN/FP) 6/19 = 31%	Prevalence of lesions positive for SFD 74/93 = 80%

**Table 4:** Performance of ventrum, cloacal and head swabs in the snakes with any positive detection. A higher agreement means that it is a better predictor of whether the snake is positive if only that swab was taken. The agreement for the sores was 80% positive. These data are from snakes near the end of hibernation in 2019 and 2020.

Performance of Ventral, Cloacal and Head Swabs on Snakes that Had Any Positive qPCR Swab.						
	Ventrum Negative	Ventrum Positive	Cloaca Negative	Cloaca Positive	Head Negative	Head Positive
One year old (n = 15) ^a^	7	5 ^b^	9	3 ^b^	9	4 ^b^
Percent positivity		42%		25%		31%
Older snakes (n = 54)	23	31	19	34	39	15
Percent positivity		57%		64%		28%

^a^ = three snakes in this group did not have complete sample results. ^b^ = ventral was the sole positive in two snakes; cloaca was the sole positive in one snake; and head was the sole positive in one snake.

## Data Availability

Data are contained within the article.

## References

[1] Skerratt I.F., Berger L., Speare R., Cashins S., McDonald K.R., Phillott A.D., Hines H.B., Kenyon N. (2007). Spread of Chytridiomycosis has caused the rapid global decline and extinction of frogs. EcoHealth.

[2] Martel A., Spitzen-van der Sluijs A., Blooi M., Bert W., Ducatelle R., Fisher M.C., Woeltjes A., Bosman W., Chiers K., Bossuyt F. (2013). *Batrachochytrium salamandrivorans* sp. nov. causes lethal chytridiomycosis in amphibians. Proc. Natl. Acad. Sci. USA.

[3] Hoyt J.R., Langwig K.E., White J.P., Kaarakka H.M., Redell J.A., Kurta A., DePue J.E., Scullon W.H., Parise K.L., Foster J.T. (2018). Cryptic connections illuminate pathogen transmission within community networks. Nature.

[4] Allender M.C., Raudabaugh D.B., Gleason F.H., Miller A.N. (2015). The natural history, ecology, and epidemiology of *Ophidiomyces ophiodiicola* and its potential impact on free-ranging snake populations. Fungal Ecol..

[5] Clark R.W., Marchand M.N., Clifford B.J., Stechert R., Stephens S. (2011). Decline of an isolated timber rattlesnake (*Crotalus horridus*) population: Interactions between climate change, disease, and loss of genetic diversity. Biol. Conserv..

[6] Di Nicola M.R.D., Coppari L., Notomista T., Marini D. (2022). *Ophidiomyces ophidiicola* detection and infection: A global review on a potential threat to the world’s snake populations. Eur. J. Wildl. Res..

[7] Allain S.J.R., Duffus A.L.J., Marschang R.E. (2022). Editorial: Emerging infections and diseases of herpetofauna. Front. Vet. Sci..

[8] Allender M.C., Hileman E.T., Moore J., Tetziaff S. (2016). Detection of *Ophidiomyces ophiodiicola*, the causative agent of Snake Fungal Disease, in the Eastern Massassauga (*Sistrurus catenatus*) in Michigan. J. Wildl. Dis..

[9] Lorch J.M., Knowles S., Lankton J.S., Michell K., Edwards J.L., Kapfer J.M., Staffen R.A., Wild E.R., Schmidt K.Z., Ballmann A.E. (2016). Snake fungal disease: An emerging threat to wild snakes. Philos. Trans. R. Soc. Lond. B Biol. Sci..

[10] Latney L.T.V., Wellehan J.F.X. (2020). Selected emerging infectious diseases of squamata: An update. Vet. Clin. N. Am. Exot. Anim. Pract..

[11] Allender M.C., Baker S., Wylie D., Loper D., Dreslik M.J., Phillips C.A., Maddox C., Driskell E.A. (2015). Development of snake fungal disease after experimental challenge with *Ophidiomyces ophiodiicola* in Cottonmouths (*Agkistrodon piscivorous*). PLoS ONE.

[12] Lorch J.M., Lankton J., Werner K., Falendysz E.A., McCurley K., Blehert D.S. (2015). Experimental infection of snakes with *Ophidiomyces ophiodiicola* causes pathological changes that typify snake fungal disease. mBio.

[13] McKenzie J.M., Price S.J., Connette G.M., Bonner S.J., Lorch J.M. (2021). Effects of snake fungal disease on short-term survival, behavior, and movement of free-ranging snakes. Ecol. Appl..

[14] McKenzie C.M., Oesterle P.T., Stevens B., Shirose L., Mastromonaco G.F., Lillie B.N., Davy C.M., Jardine C.M., Nemeth N.M. (2020). Ophidiomycosis in Red Cornsnakes (*Pantherophis guttatus*): Potential roles of brumation and temperature on pathogenesis and transmission. Vet. Pathol..

[15] Lorch J.M., Price S.J., Lankton J.S., Drayer A.N. (2021). Confirmed cases of *Ophidiomyces* in museum specimens from as early as 1945, United States. Emerg. Infect. Dis..

[16] Ladner J.T., Palmer J.M., Ettinger C.L., Stajich J.E., Farrell T.M., Glorioso B.M., Lawson B., Price S.J., Stengle A.G., Grear D.A. (2022). The population genetics of the causative agent of snake fungal disease indicate recent introductions to the USA. PLoS Biol..

[17] Allender M.C., Dreslik M., Wylie S., Phillips C., Wylie D.B., Maddox C., Delaney M.A., Kinsel M.J. (2011). *Chrysosporium* sp. Infection in Eastern Massasauga rattlesnakes. Emerg. Infect. Dis..

[18] Cheatwood J.L., Jacobson E.R., May P.G., Farrell T.M., Homer B.L., Samuelson D.A., Kimbrough J.W. (2003). An outbreak of fungal dermatitis and stomatitis in a free-ranging population of pygmy rattlesnakes (*Sistrurus milarius barbouri*) in Florida. J. Wildl. Dis..

[19] Dolinski A.C., Allender M.C., Hsiao V., Maddox C.W. (2014). Systemic *Ophidiomyces ophiodiicola* Infection in a Free-Ranging Plains Garter Snake (*Thamnophis radix*). J. Herpetol. Med. Surg..

[20] Franklinos L.H.V., Lorch J.M., Bohuski E., Fernandez J.R.-R., Wright O.N., Fitzpatrick L., Petrovan S., Durrant C., Linton C., Baláž V. (2017). Emerging fungal pathogen *Ophidiomyces ophiodiicola* in wild European snakes. Sci. Rep..

[21] Baker S.J., Haynes E., Gramhofer M., Standord K., Bailey S., Christman M., Conley K., Frasca S., Ossiboff R.J., Lobato D. (2019). Case definition and diagnostic testing for snake fungal disease. Herpetol. Rev..

[22] Haynes E., Chandler H.C., Stegenga B.J., Adamovicz L., Ospinal E., Zerpa-catanhom D., Stevenson D.J., Allender M.C. (2020). Ophidiomycosis surveillance of snakes in Georgia, USA reveals new host species and taxonomic associations with disease. Sci. Rep..

[23] Paré J.A., Sigler L. (2016). An overview of reptile fungal pathogens in the genera *Nannizziopsis*, *Paranannizziopsis*, and *Ophidiomyces*. J. Herpetol. Med. Surg..

[24] McKenzie J.M., Price S.J., Fleckenstein J.L., Drayer A.N., Connette G.M., Bohuski E., Lorch J.M. (2019). Field diagnostics and seasonality of *Ophidiomyces ophiodiicola* in wild snake populations. EcoHealth.

[25] Davy C.M., Shirose L., Campbell D., Dillon R., McKenzie C., Nemeth N., Braithwaite T., Cai H., Degazio T., Dobbie T. (2021). Revisiting Ophidiomycosis (Snake fungal disease) after a decade of targeted research. Front. Vet. Sci..

[26] McCoy C.M., Lind C.M., Farrell T.M. (2017). Environmental and physiological correlates of the severity of clinical signs of snake fungal disease in populations of pygmy rattlesnakes (*Sistrurus milarius barbouri*). Conserv. Physiol..

[27] Tetzlaff S.J., Ravesi M.J., Allender M.C., Carter E.T., DeGregorio B.A., Josimovich J.M., Kinsbury B.A. (2017). Snake fungal disease affects behavior of free-ranging Massasauga rattlesnakes (*Sistrurus catenatus*). Herpetol. Conserv. Biol..

[28] Lind C.M., Agugliaro J., Lorch J.M., Farrell T.M. (2023). Ophidiomycosis is related to seasonal patterns of reproduction, ecdysis, and thermoregulatory behavior in a free-living snake species. J. Zool..

[29] Burger J., Gochfeld M., Zappalorti R., Bunnell J., Jeitner C., Schneider D., Ng K., DeVito D., Lorch J.M. (2023). Prevalence of *Ophidiomyces ophiodiicola* in free-ranging Northern Pine Snakes (*Pituophis melanoleucus melanleucus*) in New Jersey. Environ. Monit. Assess..

[30] Dillon R.M., Paterson J.E., Manorome P., Ritchie K., Shirose L., Slavik E., Davy C.M. (2022). Seasonal and interspecific variation in the prevalence of *Ophidiomyces ophidiicola* and ophidiomycosis in a community of free-ranging snakes. J. Wildl. Dis..

[31] Hileman E.T., Allender M.C., Bradke D.R., Faust L.J., Moore J.A., Ravesi M.J., Tetzlaff S.J. (2018). Estimation of *Ophidiomyces* prevalence to evaluate snake fungal disease risk. J. Wildl. Manag..

[32] Glorioso B.M., Waddle J.H., Green D.E., Lorch J.M. (2016). First documented case of snake fungal disease in a free-ranging wild snake in Louisiana. Southeast. Nat..

[33] Marini D., Di Nicola M.R., Crocchianti V., Notomista T., Iversen D., Coppari L., Di Criscio M., Brouard V., Dorne J.-L.C.M., Rüegg J. (2023). Pilot survey reveals ophidiomycosis in dice snakes *Natrix tessellata* from Lake Garda, Italy. Vet. Res. Commun..

[34] Blanvillain G., Lorch J.M., Joudrier N., Bury S., Cuenot T., Franzen M., Martínez-Freiría F., Guiller G., Halpern B., Kolanek A. (2023). Contribution of host species and pathogen clade to snake fungal disease hotspots in Europe. bioRxiv.

[35] Burger J., Zappalorti R.T. (2011). The Northern Pine Snake (*Pituophis melanoleucus*) in New Jersey: Its Life History, Behavior and Conservation. Reptiles: Biology, Behavior, and Conservation.

[36] Burger J., Zappalorti R.T. (2016). Conservation and protection of threatened Pine Snakes (*Pituophis melanoleucus*) in the New Jersey Pine Barrens USA. Herpetol. Conserv. Biol..

[37] Burger J., Zappolorti B., Gochfeld M., Burket D., Schneider D., McCort M., Jeitner C. (2012). Long-term use of hibernaculum by Northern Pine Snakes (*Pituophis melanoleucus*). J. Herpetol..

[38] Burger J., Zappalorti R.T. (2015). Hibernation site philopatry in Northern Pine Snakes (*Pituophis melanoleucus*) in New Jersey. J. Herpetol..

[39] Burger J., Zappalorti R.T., Gochfeld M. (2018). Hatchling survival to breeding age in Northern Pine Snakes (*Pituophis melanoleucus*) in the New Jersey Pine Barrens: Human effects on recruitment from 1986 to 2017. PLoS ONE.

[40] Bohuski E., Lorch J.M., Griffin K.M., Blehert D.S. (2015). TaqMan real-time polymerase chain reaction for detection of *Ophidiomyces ophiodiicola*, the fungus associated with snake fungal disease. BMC Vet. Res..

[41] Siegel S. (1956). Nonparametric Statistics.

[42] (2020). Statistical Analysis Systems (SAS) 2020.

[43] McDonald J.H. (2022). Fisher’s Exact Test of Independence. Handbook of Biological Statistics—On Line. https://www.biostathandbook.com/fishers.html#:~:text=Fisher%27s%20exact%20test%20is%20more,test%20for%20larger%20sample%20sizes.

[44] Korevaar D.A., Gopalakrishna G., Cohen J.F., Bossuyt P.M. (2019). Targeted test evaluation: A framework for designing diagnostic accuracy studies with clear study hypotheses. Diagn. Progn. Res..

[45] Lind C.M., Lorch J.M., Moore I.T., Vernasco B.J., Farrell T.M. (2018). Seasonal sex steroids indicate reproductive costs associated with snake fungal disease. J. Zool..

[46] Duffus A.L.J., Hughes D.F., Kautz A., Allain S.J.R., Meshaka W.E. (2022). Repeated sampling of wild individuals reveals *Ophidiomyces ophidiicola* infection dynamics in a Pennsylvania snake assemblage. J. Wildl. Dis..

[47] Fuchs L.D., Tupper T.A., Aguillar R., Lorentz E.B., Bozarth C.A., Fernandez D.J., Lawlor D.M. (2020). Detection of *Ophidiomyces ophidiicola* at two mid-Atlantic natural areas in Anne Arundel County, Maryland and Fairfax County, Virginia, USA. Amph. Rept. Conserv..

[48] Chandler H.C., Allender M.C., Stegenga B.S., Haynes E., Ospina E., Stevenson D.J. (2019). Ophidomycosis prevalence in Georgia’s Eastern indigo snake (*Drymarchon couperi*) populations. PLoS ONE.

[49] Vivirito K., Haynes E., Adamovicz L., Wright A., Durante K., Stanford K., Scott E., Allender M. (2021). Ultraviolet fluorescence as a field-applicable screening tool for lesions consistent with Ophidiomycosis in Lake Erie Watersnakes (*Nerodia sipedon insularum*). J. Wildl. Dis..

[50] Campbell L.J., Burger J., Zappalorti R.T., Bunnell J.F., Winzeler M.E., Taylor D.R., Lorch J.M. (2021). Soil reservoir dynamics of *Ophidiomyces ophidiicola*, the causative agent of snake fungal disease. J. Fungi.

[51] Burbrink F.T., Lorch J.M., Lips K.R. (2017). Host susceptibility to snake fungal disease is highly dispersed across phylogenetic and functional trait space. Sci. Adv..

[52] Rajeev S., Sutton D.A., Wickes B.L., Miller D.L., Giri D., Van Meter M., Thompson E.H., Rinaldi M.G., Romanelli A.M., Cano J.F. (2009). Isolation and characterization of a new fungal species, *Chrysosporium ophiodiicola*, from a mycotic granuloma of a black rat snake (*Elaphe obsoleta obsoleta*). J. Clin. Microbiol..

